# Systemic Inflammation Increases the Susceptibility to Levodopa-Induced Dyskinesia in 6-OHDA Lesioned Rats by Targeting the NR2B-Medicated PKC/MEK/ERK Pathway

**DOI:** 10.3389/fnagi.2020.625166

**Published:** 2021-02-01

**Authors:** Aijuan Yan, Lu Song, Yu Zhang, Xijin Wang, Zhenguo Liu

**Affiliations:** Department of Neurology, Xinhua Hospital Affiliated to Shanghai Jiao Tong University School of Medicine, Shanghai, China

**Keywords:** L-dopa induced dyskinesia, Parkinson's disease, systemic inflammation, NR2B, neuroinflammation

## Abstract

**Background:** The long-term administration of levodopa (L-dopa), the gold-standard treatment for Parkinson's disease (PD), is irreparably associated with L-dopa-induced dyskinesia (LID), which dramatically affects the quality of life of patients. However, the underlying molecular mechanisms of how LID exacerbates remain unknown. Neuroinflammation in the striatum plays an active role in LID. These findings prompt an investigation of non-neuronal mechanisms of LID. This study will examine the effects of systemic inflammation in the development and progression of LID.

**Methods:** To evaluate the possible influence of systemic inflammation in the appearance of LID, the PD rats received an intraperitoneal (IP) injection of various concentrations of lipopolysaccharides (LPS, 1, 2, and 5 mg/kg) or saline. One day later, these PD rats started to receive daily treatment with L-dopa (6 mg/kg) along with benserazide (6 mg/kg) or saline for 21 days, and dyskinesia was evaluated at several time points. Moreover, the activation of microglia and astrocytes and the molecular changes in NR2B and mGLUR5 signaling pathways were measured.

**Results:** We found that systemic inflammatory stimulation with LPS exacerbated the intensity of abnormal involuntary movements (AIMs) induced by L-dopa treatment in 6-hydroxydopamine (6-OHDA) lesioned rats. The LPS injection activated the gliocytes and increased the levels of proinflammatory cytokines in the striatum in LID rats. The PD rats that received the LPS injection showed the overexpression of p-NR2B and NR2B, as well as activated PKC/MEK/ERK and NF-κB signal pathways in response to the L-dopa administration. On the contrary, clodronate-encapsulated liposomes (Clo-lipo), which could suppress the inflammatory response induced by peripheral LPS injection, improved behavioral dysfunction, inhibited neuroinflammation, prevented NR2B overexpression, and decreased the phosphorylation of PKC/MEK/ERK and NF-κB signaling pathways.

**Conclusion:** This study suggests that systemic inflammation, by exacerbating preexisting neuroinflammation and facilitating NR2B subunit activity, may play a crucial role in the development of LID. The administration of Clo-lipo restores the effects of LPS and decreases the susceptibility to LID in 6-OHDA lesioned rats.

## Introduction

Parkinson's disease (PD) is characterized by the progressive loss of dopaminergic neurons in the substantia nigra (SN) and leads to bradykinesia, rigidity, and tremor (Kalia and Lang, 2015). Levodopa (L-dopa), the gold-standard treatment for PD, alleviates the motor symptoms of PD. However, its long-term administration is irreparably associated with abnormal involuntary movements (AIMs), termed L-dopa-induced dyskinesia (LID) (Espay et al., 2018), which dramatically affects the quality of life of patients. Treatment of high doses of L-dopa and the severity of PD mainly contributed to the development and progression of dyskinesia (Cilia et al., 2014).

Recently, non-neuronal mechanisms have been proposed to play important roles in the development of LID (Del-Bel et al., 2016). The immune system has emerged as an important player in LID and was a potential target for pharmacological therapy (Pisanu et al., 2018). Glial cells are significant in the immune reaction of the brain to noxious insults by establishing the inflammatory milieu surrounding activated glial cells (Bortolanza et al., 2015a). Previous researches showed that the L-dopa chronic treatment eliciting AIMs were associated with microgliosis, astrocytosis, and an increased expression of proinflammatory cytokines (Bortolanza et al., 2015a; Del-Bel et al., 2016; Carta et al., 2017). The study of Mulas et al. (2016) showed that a pulsatile treatment with L-dopa resulted in a neuroinflammatory response in the striatum, while continuous delivery of the drug was void of any inflammatory response and dyskinetic outcome. Neuroinflammation is a shared feature of LID in the human brain and in animal models. Conditions that induce neuroinflammatory response and cytokine release could facilitate LID (Mulas et al., 2016). Several reports revealed that peripheral inflammatory response could induce neuroinflammation *via* the activation of microglia and the production of proinflammatory cytokines, including interleukin-6 (IL-6), interleukin-1β (IL-1β), and tumor necrosis factor-α (TNF-α) in the brain (Murta et al., 2015). A well-studied model of systemic inflammation in rodents is the intraperitoneal (IP) injection of *Escherichia coli* lipopolysaccharide (LPS) from Gram-negative bacteria to induce the innate immune response (Wu et al., 2012). Whether the systematic inflammatory response is involved in LID is unclear.

N-methyl-D-aspartate (NMDA) is a heterotetramer ion channel assembled from a combination of GluN1, GluN2, and GluN3 subunits (Yang et al., 2018). NR2A and NR2B are the most common subtypes of NMDA receptors (NMDARs) found in the central nervous system (CNS) of mammals. Neuronal apoptosis is prevented when NR2A is selectively activated, whereas the activation of NR2B is associated with the inflammatory response (Zhang et al., 2018). NR2B is best characterized as a regulatory subunit that plays an important role in the inflammatory response (Chen et al., 2008). The proinflammatory cytokines in the brain have been shown to facilitate the NR2B subunit of the NMDA receptor activity (Viviani et al., 2003). The phosphorylation of the NR2B subunit plays a very important role in the regulation of NMDA receptor function and has been connected to alterations of synaptic efficacy of the NMDA receptor. CP-101,606, an NR2B-selective antagonist, has been reported to reduce dyskinesia as measured by clinical scoring (Ba et al., 2019). A possible role for the NR2B receptor in dyskinesia in patients with PD (Herring et al., 2017) or Parkinsonian animals (Gan et al., 2014) has been highlighted. However, whether the activation of NR2B in the striatum plays a contributing role in the exacerbation of LID susceptibility after systemic inflammation is currently unknown.

How systematic inflammation aggravated LID remains unclear. Based on the above evidence, we proposed that the systematic inflammatory response, through its actions on NR2B, would influence the development of LID. Furthermore, based on the existence of a correlation between LID and NR2B receptor-dependent signaling pathways, we performed the biochemical analysis in order to elucidate the molecular mechanisms of LPS on LID. To achieve systematic inflammation, we used a model of IP injection of LPS in 6-hydroxydopamine (6-OHDA)-lesioned rats. This study will discuss how systematic inflammation affects LID in 6-OHDA lesioned rats.

## Materials and Methods

### Animals

Adult male Sprague-Dawley rats in Specific Pathogen Free (SPF) grade (mean weight 200–250 g) were purchased from the Shanghai Laboratory Animal Center (SLAC; Shanghai, China). The production license number is SCXK 2017-0005. The rats were housed five per cage under a temperature of 22.0 ± 2.0°C and humid conditions, in a 12 h-light/dark cycle with free access to food and water. All animal works were performed in accordance with the guidelines of the National Institutes of Health for the care and use of laboratory animals as stipulated by the Institutional Animal Care and Use Committee of the School of Medicine, Shanghai Jiao Tong University, Shanghai, China. All experimental animal procedures followed the Animal Research: Reporting of *In Vivo* Experiments (ARRIVE) guidelines (https://www.nc3rs.org.uk/arrive-guidelines), and the guidelines of the Regulation for the Administration of Affairs Concerning Experimental Animals of China enacted in 1988.

### Rat Model of PD

6-hydroxydopamine-lesioned PD rat models were performed according to our previous study (Xie et al., 2014). Briefly, rats were anesthetized with ketamine (100 mg/kg) by IP injection. Then, the rats received 6-OHDA (16 μg dissolved in 4 μl of 0.9% saline containing 0.2% L-ascorbic acid; Sigma-Aldrich, St. Louis, MO, USA) injection into the right middle forebrain bundle (MFB) at the following coordinates relative to the bregma: (1) anterior-posterior (AP), 3.7 mm; medial-lateral (ML), 1.7 mm; and dorsal-ventral (DV), 7.8 mm and (2) AP, 4.4 mm; ML, 1.2 mm; and DV, 7.8 mm. The injection speed was 1 μl/min. The needle was withdrawn 5 min after infusion. After 21 days, rats exhibiting >7 contralateral turns over 1 min in response to IP injections of apomorphine (0.5 mg/kg) were selected as successful PD rat models and could be used for subsequent experiments.

### Drug Treatment and LID

**Figure 1:**
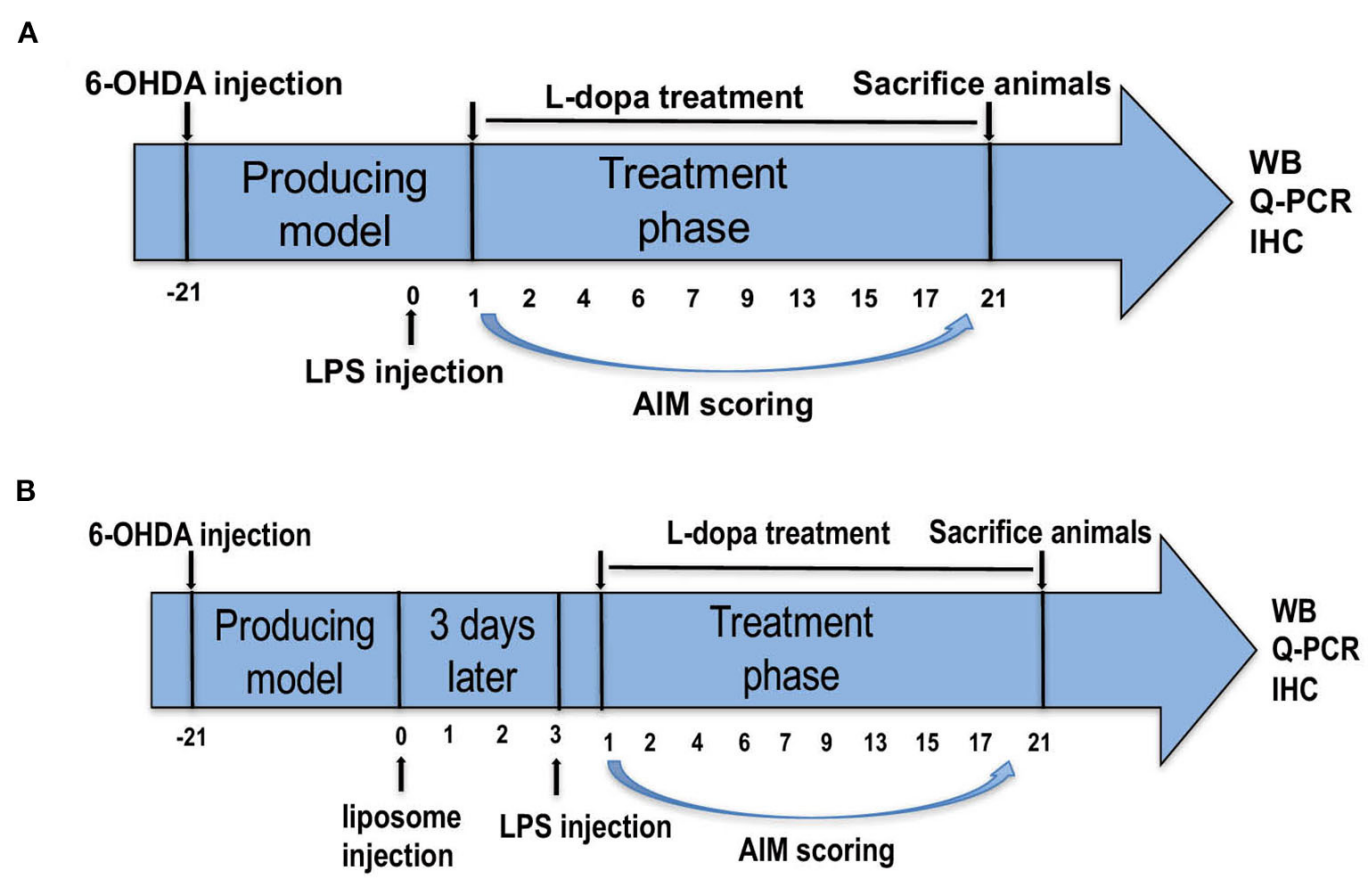
Experimental design of the study. **(A)** Parkinson's disease (PD) rats were unilaterally injected with 6-hydroxydopamine (6-OHDA) in the middle forebrain bundle (MFB). Contralateral turning behaviors after apomorphine injection were tested. Rats in the PD+LPS+L-dopa group were administrated with lipopolysaccharide (LPS). Twenty four hours later, rats in the PD+saline+L-dopa and PD+LPS+L-dopa group were administrated with L-dopa (6 mg/kg, IP) plus benserazide [6 mg/kg, intraperitoneal (IP)] for 21 days. Abnormal involuntary movements (AIMs) were evaluated during this period, at days 1, 2, 4, 6, 7, 9, 13, 15, 17, and 21. **(B)** The successful PD rats were divided into two groups and received IP injections of phosphate-buffered saline (PBS) liposomes (1 ml/100 g) or clodronate-liposomes (Clo-lipos, 1 ml/100 g), respectively. After 3 days, the animals received a single injection of LPS (2 mg/kg, IP) and were then treated with L-dopa/benserazide (6/6 mg/kg, IP) once daily for 21 days. The animals were sacrificed 2 h after the last injection for Western blot, quantitative PCR (q-PCR), and immunofluorescence.

In the first part of our study, the successful PD rats were divided into four groups randomly. Systemic inflammation was induced by one injection of LPS from *E. coli* O26:B6 (Sigma, St. Louis, MO, USA). These PD rats received a single IP injection of LPS (1, 2, and 5 mg/kg; Sigma-Aldrich Chemical, M O, USA; Cho et al., 2015; Ho et al., 2015; Kosyreva et al., 2018) or 0.9% saline. After 24 h, they were administered with L-dopa (6 mg/kg, IP.) along with benserazide (6 mg/kg, i.p.) once daily for 21 days to induce a model of dyskinesia (Tronci et al., 2017; Figure 1A).

Clodronate-encapsulated liposomes (Clo-lipo) were widely used to deplete peripheral macrophages (Ma et al., 2016; Yan et al., 2018). Clodronate was encapsulated in liposomes with a concentration of 5 mg clodronate/ml, a concentration that ensures the depletion of 80% of macrophages within 72 h after systemic administration (Seiler et al., 1997). Our study found that Clo-lipo can deplete 82% of macrophages in the spleen 72 h after injection (Supplementary Figure 1). In the second part, the successful PD rats received IP injections of phosphate-buffered saline (PBS) liposomes (1 ml/100 g of the order preparation; Nico van Rooijen; Amsterdam, The Netherlands) or Clo-lipo (1 ml/100 g of the order preparation; Nico van Rooijen; Amsterdam, The Netherlands). After 72 h, the two rat groups were administered with LPS (2 mg/kg, IP) and then treated with an administration of L-dopa/benserazide (6/6 mg/kg, IP) once daily for 21 days (Figure 1B). Two hours after the final L-dopa/benserazide treatment, the rats were sacrificed by CO_2_ euthanization, and brain samples were stored at −80°C until analysis.

### Abnormal Involuntary Movement Ratings

Abnormal involuntary movements were evaluated according to the rat dyskinesia scale described in our previous publications (Gan et al., 2015). On days 1, 2, 4, 6, 9, 13, 17, and 21 of the L-dopa/benserazide treatment, AIMs were individually observed in the 6-OHDA-lesioned PD rats after the L-dopa/benserazide injection by an experimenter who was unaware of the pharmacological intervention. Rats were placed individually in plastic cages and observed for 1 min at 20 min intervals for a duration of 120 min. AIMs were classified into three subtypes according to their distribution as axial movement, limb movement, and orolingual movement. The severity of each AIM subtype was assessed using scores from 0 to 4 (0: absent, 1: present <50% of the time; 2: present >50% of the time; 3: present all the times but interrupted by external stimuli; and 4: present all the times, and not interrupted by external stimuli). For every rat, the ALO (Axical, Limb, Orolingual) AIMs score was calculated by adding each of the three individual scores.

### Immunohistochemistry

Immunohistochemical staining of rat brain sections was performed as previously described (Xie et al., 2014). The brains were removed, postfixed for 24 h in 4% paraformaldehyde (PFA), and then cryoprotected in 30% sucrose in PBS (pH 7.4). Subsequently, serial coronal sections (cut thickness: 20 μm) were cut on a freezing microtome. The brain sections were fixed with 4% PFA for 15 min and then incubated in PBS for 10 min. Slides were blocked for 1 h in 10% normal donkey serum and then incubated in a primary antibody at 4°C overnight. Primary antibodies included rabbit anti-iba1 (1:200, Abcam) and rabbit anti-glial fibrillary acidic protein (GFAP; 1:400, Abcam). After being washed three times with PBS, the sections were incubated with the corresponding donkey anti-rabbit secondary antibody conjugated to Alexa Fluor 594 (1:400 dilution, Life Technologies) for 1 h at room temperature. The brain sections were viewed using a fluorescence microscope (Leica, Solms, Germany). Three fields measuring 150 × 150 μm in the lesioned striatum were imaged in each section. A total of 10 sections (taking every other section spaced 200 μm apart) were evaluated for each rat as previously described (Ma et al., 2016). The mean integrated optical density (IOD) was measured by the ImageJ software (Media Cybernetics, Bethesda, MO). The ratio of the mean IOD between the different groups was used for further analysis.

### Quantitative PCR

The rats from each group were sacrificed by anesthetic overdose 21 days after L-dopa injection. Total RNA was isolated from rat brains using Trizol Reagent (Life Technologies, Rockville, MD, USA) and was reverse transcribed to cDNA using the PrimeScript RT Reagent kit (Takara Bio, Inc., Otsu, Japan). qPCR was performed using an SYBR Green kit (Takara Bio, Inc., Otsu, Japan) according to the manufacturer's instructions. The outcome was expressed as the fold-difference normalized to glyceraldehyde-3-phosphate dehydrogenase (GAPDH). Q-PCR was detected using an ABI PRISM 7500 Sequence Detection system (Thermo Fisher Scientific, Inc., Waltham, USA). The sequences of the primer pairs for proinflammatory cytokine genes are as follows:

IL-1β (F: AGCTGCACTGCAGGCTTCGAGATG, R: GAACTGTGCAGACTCAAA CTCCAC); IL-6 (F: TCCTACCCCAACTTCCAATGCTC, R: TTGGATGGTCTTGGTCCTTAGCC); TNF-α (F: CACGCTCTTCTGTCTACTG AACTTCG, R: TGCTCC TCCGCTTGGTGGTT); and GAPDH (F: TTCCTACC CCCAATGTATCCG, R: CATGAGGTCCACCACCCTGTT).

### Western Blotting

Tissue samples were prepared by homogenizing the brain in a standard lysis buffer. The protein concentration was measured using a Pierce BCA Protein Assay Kit (ThermoFisher Scientific). Forty micrograms of protein per well were separated by Sodium dodecyl sulphate polyacrylamide gel electrophoresis (SDS-PAGE) and transferred onto a nitrocellulose membrane. The membranes were blocked with 5% non-fat dried milk for 1 h and incubated with primary antibodies against iba1 (1:1,000, Abcam), GFAP (1:2,000, Abcam), p-NR2B (1:1,000, Abcam), NR2B (1:1,000, Abcam), mGLUR5 (1:1,000, Abcam), p-PKC (1:1,000, Cell Signaling Technology), PKC (1:1,000, Cell Signaling Technology), p-MEK (1:1000, Cell Signaling Technology), MEK (1:1,000, Cell Signaling Technology), p-pERK (1:1,000, Cell Signaling Technology), ERK (1:1,000, Cell Signaling Technology), and β-actin (1:1,000, Cell Signaling Technology) overnight at 4°C. After being washed with Tris-buffered saline with TWEEN 20 (TBST) buffer, the membranes were incubated with horseradish peroxidase (HRP)-conjugated secondary antibody (1:2,000, Cell Signaling Technology) for 1 h at room temperature and then subjected to chemiluminescent detection according to the manufacturer's instructions (Millipore).

### Statistical Analysis

Behavioral data were non-parametric and analyzed using the Kruskal–Wallis test followed by the Dunn's test for multiple comparisons in the case of comparing data over multiple days, or the Mann–Whitney *U*-test. Group comparisons were performed using one-way ANOVA with a Tukey's multiple comparisons test. We reported actual values of *p* from the ANOVA. All values are presented in the study as means ± SEM. Values of *p* < 0.05 were considered statistically significant. The statistical analysis was performed using the GraphPad software (GraphPad Software, Inc., La Jolla, USA).

## Results

### Effect of Peripheral LPS Injection on LID

**Figure 2:**
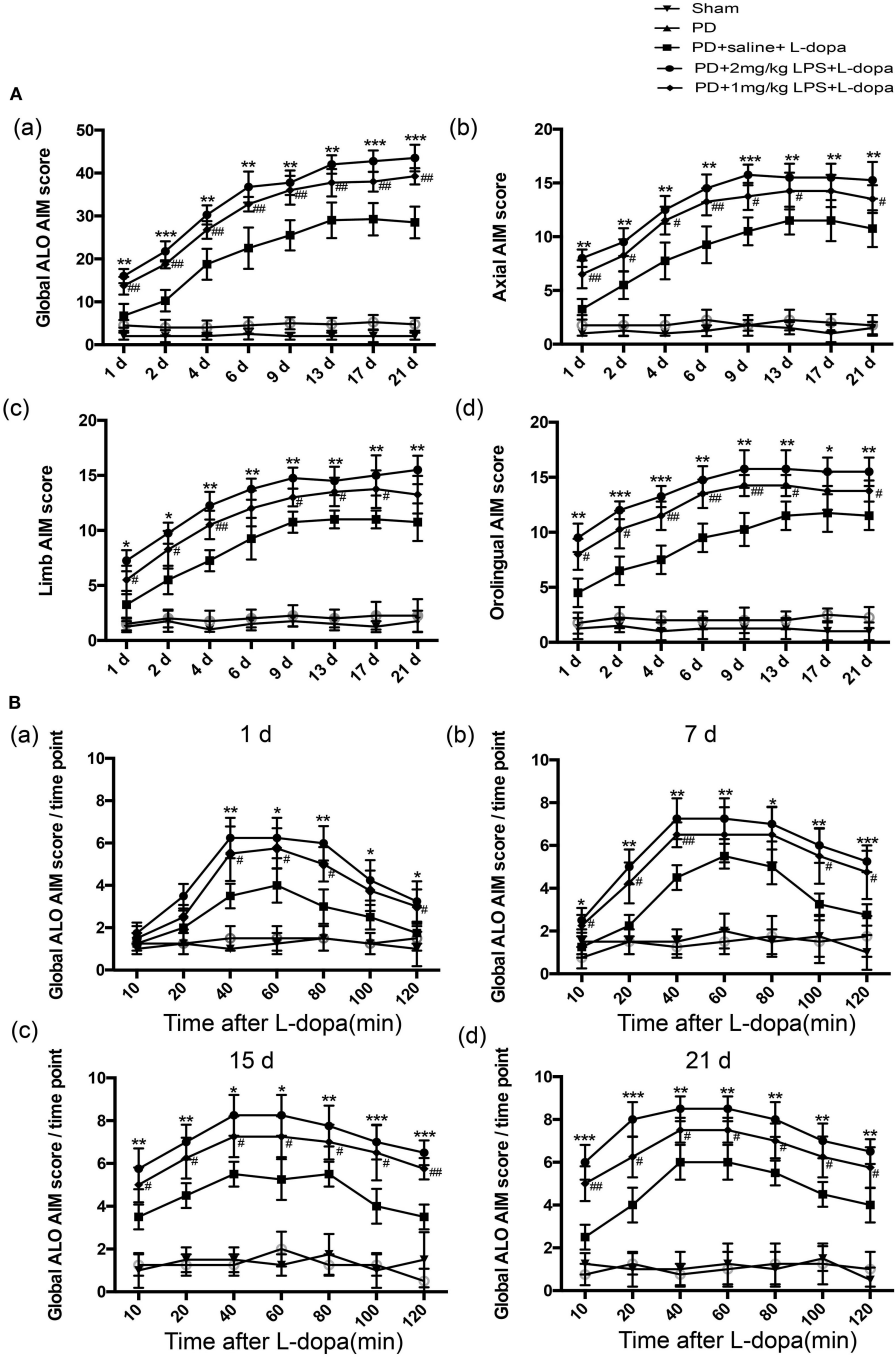
Effect of peripheral LPS injection on the ALO AIMs during a 3-week treatment with L-dopa. Rats were rated for ALO AIMs for 120 min post injection. **(A)** (a) sum of axial, limb, and orolingual, (b) axial, (c) limb, and (d) orolingual AIMs. **(B)** the global ALO AIM score at different time points at 1 day (a), 7 days (b), 15 days (c), and 21 days (d). Values are presented as the mean ± SEM. *p < 0.05, **p < 0.01, and ***p < 0.001: PD+2 mg/kg LPS+L-dopa vs. PD+saline+L-dopa; ^#^*p* < 0.05 and ^##^*p* < 0.01: PD+1 mg/kg LPS+L-dopa vs. PD+saline+L-dopa.

To evaluate whether systematic inflammation can produce an effect on dyskinesia in LID, the successful PD rats received a single IP injection of LPS used at 1 mg/kg, 2 mg/kg, and 5 mg/kg. A lower dose of L-dopa (6 mg/kg, plus 6 mg/kg benserazide, IP) was used in an attempt to better highlight the differences in the AIM score between LPS- and saline-treated PD rats during the L-dopa treatment (Tronci et al., 2017). Our results revealed that 5 mg/kg LPS resulted in the death of PD rats. The sham and PD groups received the saline for 21 days, and these rats did not develop LID. Administration of L-dopa for 21 days induced dyskinesia in both LPS and saline-treated PD rats. In terms of the ALO AIM score, PD+2 mg/kg LPS and PD+1 mg/kg LPS rats were obviously increased compared with the PD+saline rats at the same time point during the course of the L-dopa treatment (^*^p < 0.05, ^**^p < 0.01, ^***^p < 0.001 vs. PD+saline+L-dopa. ^#^*p* < 0.05, ^##^*p* < 0.01, vs. PD+saline+L-dopa, Figure 2Aa, *n* = 12/group). This was the same trend in axial AIM (Figure 2Ab), limb AIM (Figure 2Ac), and orolingual AIM (Figure 2Ad). On the L-dopa treatment days 1, 7, 15, and 21, we also analyzed the ALO AIM score of these five groups at 10, 20, 40, 60, 80, 100, and 120 min after the L-dopa treatment. Interestingly, since day 7 of using L-dopa, an increased ALO AIM score was observed in the LPS-treated rats at 10 min after the L-dopa treatment as compared with the PD+saline+L-dopa group (^*^p < 0.05, ^**^p < 0.01, ^***^p < 0.001 vs. PD+saline+L-dopa. ^#^*p* < 0.05, ^##^*p* < 0.01, vs. PD+saline+L-dopa; Figure 2B, *n* = 12/group), indicating that LPS exacerbates LID. A higher dose of LPS (2 mg/kg) had a greater effect on LID than the lower dose (1 mg/kg). These data suggested that peripheral LPS injection into PD rats showed a higher susceptibility to develop dyskinesia as compared to the saline-treated PD rats during the L-dopa treatment.

### Peripheral LPS Injection Produces Long-Lasting Effects on Neuroinflammation in the Striatum of LID Rats

**Figure 3:**
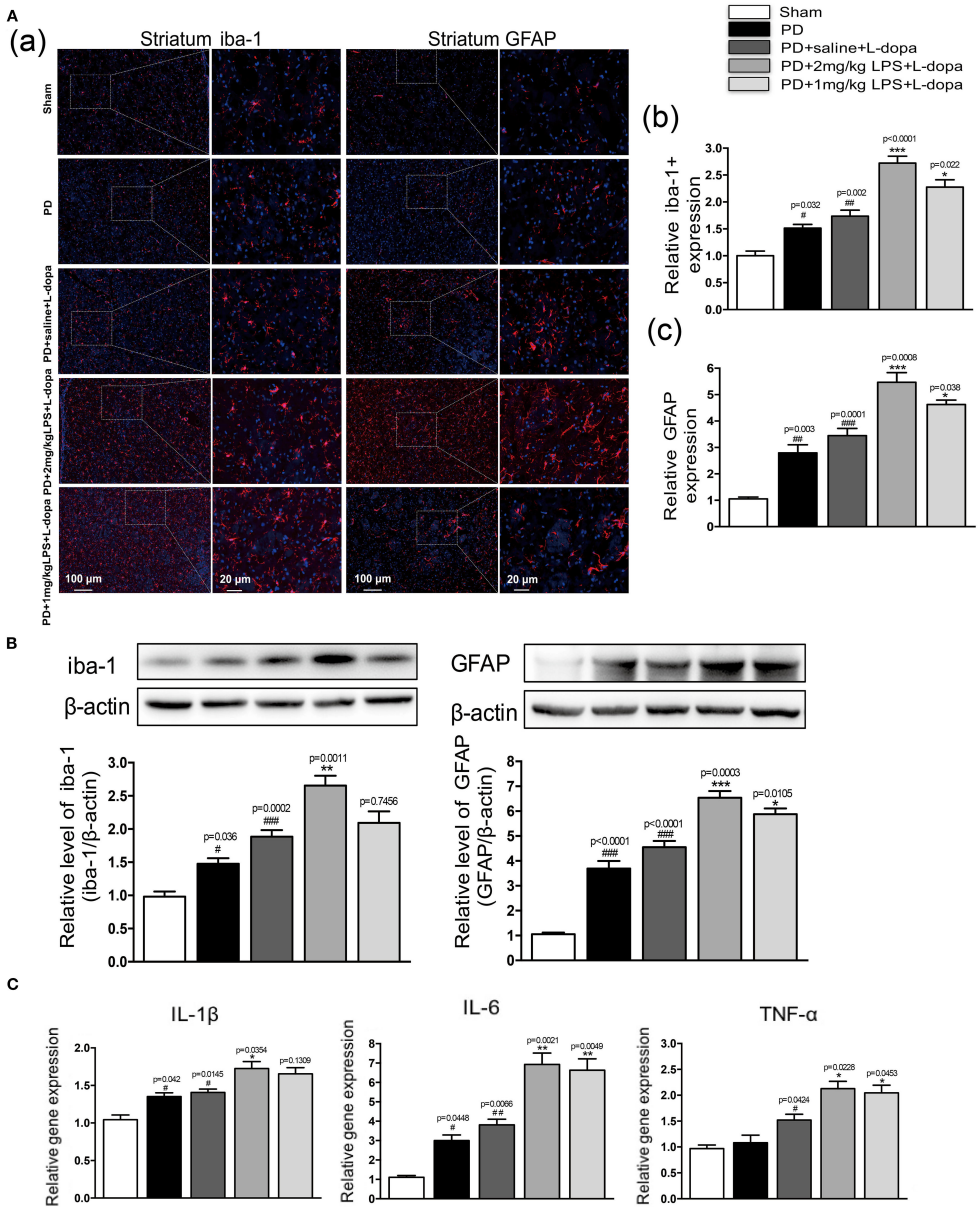
Effect of peripheral LPS injection on the striatal neuroinflammation after the pulsatile L-dopa treatment. **(A)** (a) Microglia and astrocytes were determined in the striatum by immunohistochemical analysis of Iba1 and GFAP. Scale bar = 100 μm in left panels and 20 μm in right panels. (b) The quantity of microglia in the striatum was quantified by the intensity of iba-1+immunofluorescence. (c) The number of astrocytes in the striatum was quantified by the intensity of GFAP+immunofluorescence. **(B)** Expression levels of iba-1 and GFAP were analyzed by Western blotting. **(C)** The mRNA levels of the proinflammatory mediators IL-1β, IL-6, and TNF-α normalized to GAPDH in the striatum. Values are presented as the mean ± SEM. *n* = 4 per group. ^#^*p* < 0.05, ^##^*p* < 0.01, and ^###^*p* < 0.001 vs. sham. *p < 0.05, **p < 0.01, and ***p < 0.001 vs. PD+saline+L-dopa.

To investigate if only a single IP injection of LPS is involved in neuroinflammation in normal rats, the inflammatory response in the striatum was tested. Our study found that LPS could significantly increase the expression of glia markers Iba-1 (Supplementary Figure 2Aa,b) and GFAP (Supplementary Figure 2Aa,c) and the mRNA levels of IL-1β, IL-6, and TNF-α (Supplementary Figure 2B) in the striatum of 24 h after injecting the normal rats. Furthermore, we investigated the effects of one IP injection of LPS on neuroinflammation in the striatum of LID rats. As shown in Figure 3, in the PD+saline+L-dopa group, the microglia and astrocytes in the lesioned hemispheres were activated as compared to the PD group and the sham group (^#^*p* < 0.05, ^##^*p* < 0.01 vs. sham, *n* = 4/group). To characterize the effect of peripheral LPS injection on neuroinflammation in the striatum, the activation of microglia and astrocytes, IL-1β, IL-6, and TNF-α mRNA levels were quantified. The PD rats pretreated with a challenge with LPS showed an increase of Iba1+ and GFAP+ cells in the lesioned striatum as compared to the PD+saline+L-dopa group after the last L-dopa treatment, (^*^p < 0.05, ^**^p < 0.01, ^***^p < 0.001 vs. PD+saline+L-dopa, Figures 3A,B, *n* = 4/group). Moreover, the Q-PCR analysis of mRNA for the proinflammatory cytokines was performed on day 21 of the L-dopa chronic administration. Results showed the release of peripheral LPS-infusion induced inflammatory cytokines IL-1β, IL-6, and TNF-α in the PD+LPS+L-dopa group (^*^p < 0.05, ^**^p < 0.01 vs. PD+saline+L-dopa, Figure 3C). These results showed that IP infusion of LPS induced neuroinflammation in the striatum, characterized by significant activation of microglia and astrocytes, and increased in the expression of IL-1β, IL-6, and TNF-α.

### LPS Treatments Increased the Expressions of p-NR2B and NR2B in the Striatum of LID Rats

**Figure 4:**
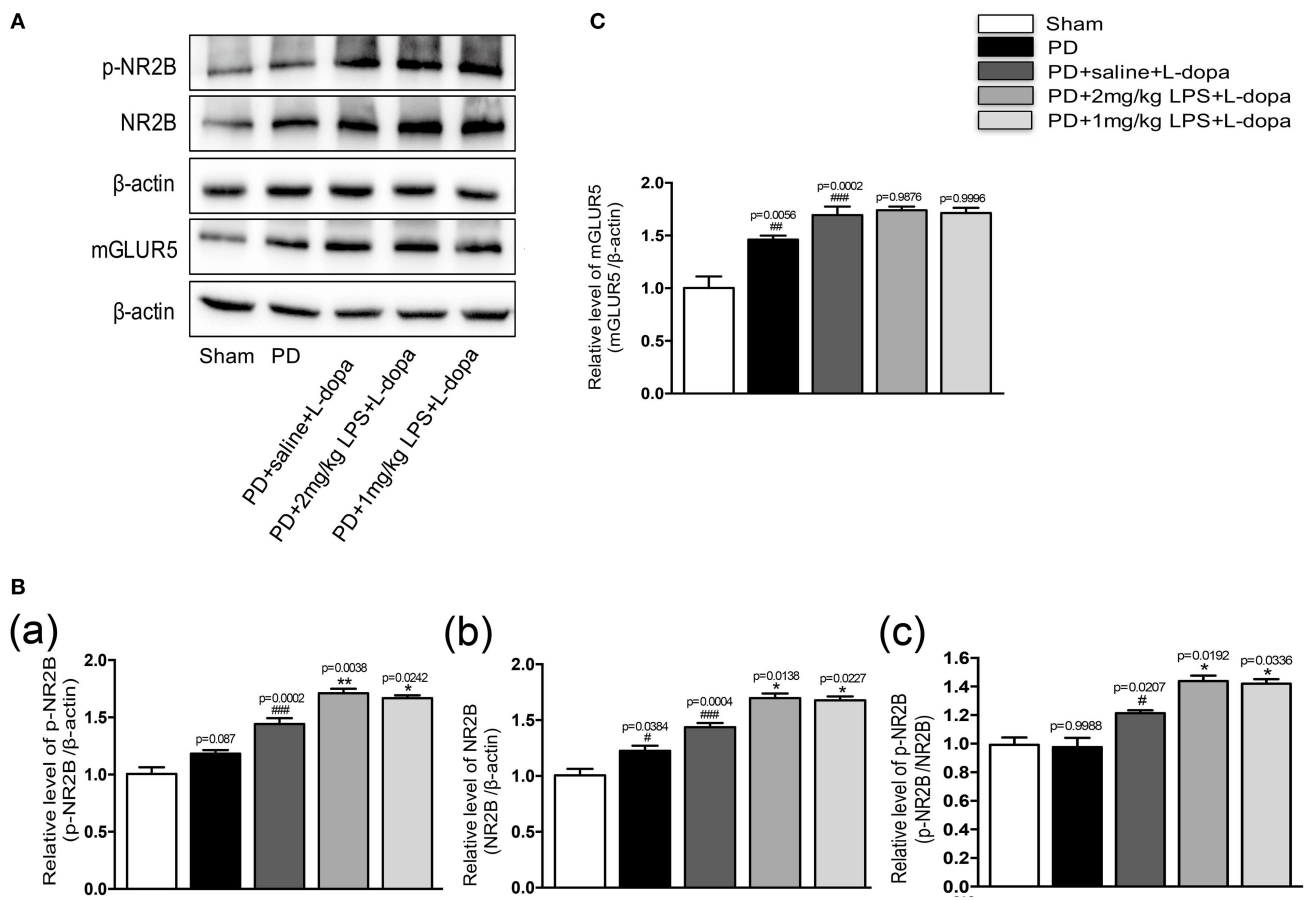
Effect of peripheral LPS injection on the expression of NR2B and mGluR5 in the striatum of LID rats. **(A)** Expression levels of p-NR2B, NR2B, and mGLUR5 in the striatum were analyzed by Western blotting. **(B)** (a) Quantification of the densitometric value of the p-NR2B protein bands is shown, normalized to β-actin. (b) Quantification of the densitometric value of the NR2B protein bands is shown, normalized to β-actin. (c) Quantification of the densitometric value of the p-NR2B protein bands is shown, normalized to NR2B. **(C)** Quantification of the densitometric value of the mGLUR5 protein bands is shown, normalized to β-actin. Values are presented as mean ± SEM. *n* = 4 per group. ^#^*p* < 0.05, ^##^*p* < 0.01, and ^###^*p* < 0.001 vs. sham. *p < 0.05, **p < 0.01 vs. PD+saline+L-dopa.

Our study found that LPS-treated rats showed significantly higher levels of phosphorylation of NR2B and total NR2B in the striatum 24 h after the injection (Supplementary Figures 3A,B). No differences were found in the mGluR5 levels in the striatum injected with LPS compared to the saline-injected group (Supplementary Figure 3C). To examine whether a single IP injection of LPS to the PD rats influenced the protein expression of NR2B and mGluR5 in the striatum in L-dopa induced animals, we performed Western blotting on the striatum tissue collected at 2 h after the last L-dopa injection. The LPS-treated PD rats showed significantly higher levels of phosphorylation of NR2B and total NR2B, in response to L-dopa, compared with the PD+saline+L-dopa group (^*^p < 0.05 vs. PD + saline+ L-dopa, Figures 4A,B, *n* = 4/group). No difference was found in the expression of mGLUR5 levels in the lesioned striatum injected with LPS compared with the PD+saline+L-dopa group (Figure 4C; *n* = 4/group). We thought that the overexpression of the NR2B subunit induced by a single IP injection of LPS might play an important role in the induction of dyskinesia during the course of the L-dopa injection.

### Lipopolysaccharide Treatments Further Activated the Striatal PKC/MEK/ERK and NF-κB Signal Pathways in the Striatum After Pulsatile L-Dopa Treatment

**Figure 5:**
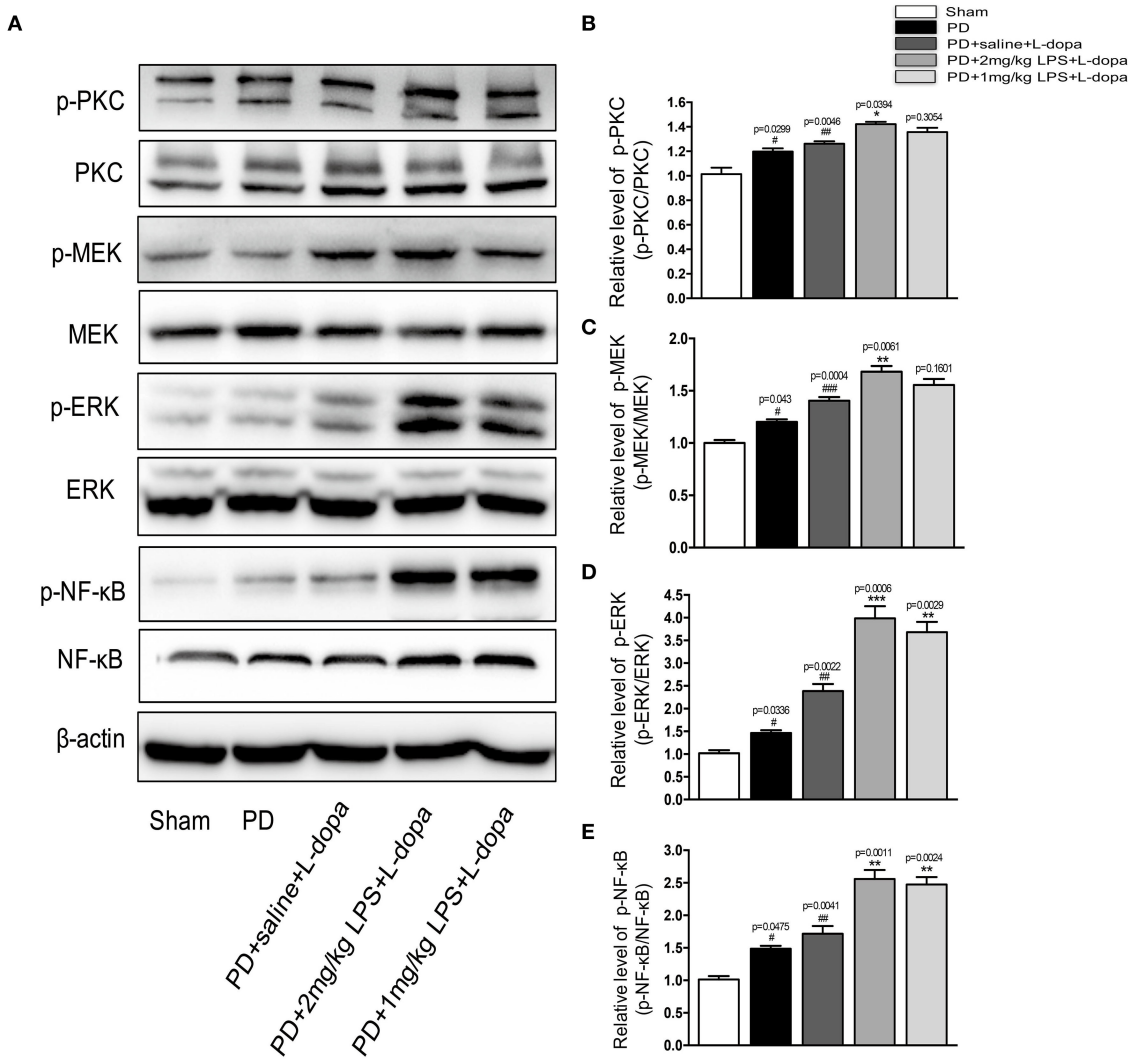
Effects of peripheral LPS injection on striatal PKC/MEK/ERK and NF-κB signaling pathways in LID rats. **(A)** Expression levels of p-PKC, PKC, p-MEK, MEK, p-ERK, ERK, p-NF-κB, and NF-κB in the striatum were analyzed by Western blotting. **(B)** Quantification of the densitometric value of the p-PKC protein bands is shown, normalized to PKC. **(C)** Quantification of the densitometric value of the p-MEK protein bands is shown, normalized to MEK. **(D)** Quantification of the densitometric value of the p-ERK protein bands is shown, normalized to ERK. **(E)** Quantification of the densitometric value of the p- p-NF-κB protein bands is shown, normalized to NF-κB. Values are presented as the mean ± SEM. *n* = 4 per group. ^#^*p* < 0.05, ^##^*p* < 0.01, and ^###^*p* < 0.001 vs. sham. *p < 0.05, **p < 0.01, ***p < 0.001 vs. PD+saline+L-dopa.

Consistent with the previous report, we found the phosphorylation of PKC/MEK/ERK was significantly increased in the lesioned striatum of the PD+saline+L-dopa group as compared to the PD group (^#^*p* < 0.05, ^##^*p* < 0.01 vs. sham, Figure 5, *n* = 4/group). Our data showed that peripheral LPS injection could increase the expression of phosphorylation of NR2B and total NR2B in the striatum of the L-dopa-treated group. Thus, the impact of LPS injection on the striatal NR2B receptor was evaluated by measuring PKC/MEK/ERK and NF-κB signal pathways. Our results demonstrated that the administration of peripheral LPS to the PD rats could significantly increase the phosphorylation of PKC/MEK/ERK and NF-κB compared with the saline-treated PD rats during the course of the L-dopa injection (^*^p < 0.05, ^***^p < 0.001 vs. PD+saline+L-dopa, Figure 5, *n* = 4/group). These results showed that a single acute intraperitoneal LPS administration to the PD rats could further activate PKC/MEK/ERK and NF-κB signaling pathways in the striatum during the L-dopa treatment.

### Clo-lipo Administration Attenuated the AIM Score and Suppressed LPS-Induced Striatal Neuroinflammation in LID Rats

**Figure 6:**
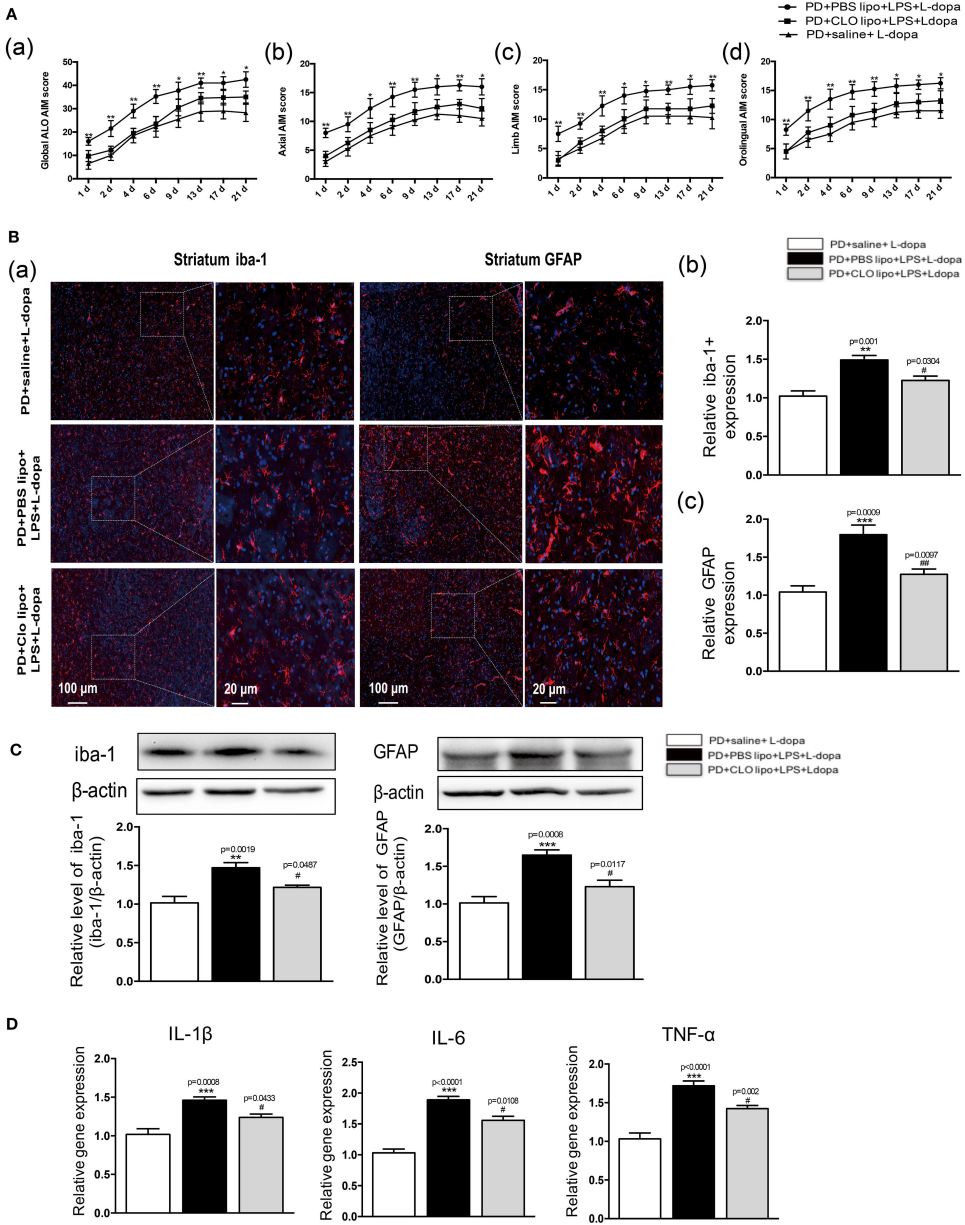
Effects of Clodronate liposome treatments on ALO AIMs score and LPS-induced activation of neuroinflammation in the striatum of LID rats. **(A)** (a) A sum of axial, limb, and orolingual, (b) axial, (c) limb, and (d) orolingual AIMs. **(B)** (a) Microglia and astrocyte were determined in the striatum by immunohistochemical analysis of Iba-1 and GFAP. Scale bar = 100 μm in left panels and 20 μm in right panels. (b) The quantity of microglia in the striatum was quantified by the intensity of iba-1+immunofluorescence. (c) The number of astrocytes in the striatum was quantified by the intensity of GFAP+immunofluorescence. **(C)** Expression levels of iba-1 and GFAP were analyzed by Western blotting. **(D)** The mRNA levels of the proinflammatory mediators IL-1β, IL-6, and TNF-α normalized to GAPDH in the striatum. Values are presented as the mean ± SEM. *n* = 4 per group. *p < 0.05, **p < 0.01, ***p < 0.001 vs. PD+saline+L-dopa. ^#^*p* < 0.05, ^##^*p* < 0.01 vs. PD+ PBS lipo+LPS+L-dopa.

A previous study has shown that this unselective macrophage depletion by Clo-lipo administration reduced the experimental acute inflammatory response induced by the LPS injection (Villaran et al., 2010; Xie et al., 2017; Pervin et al., 2018). In this study, we found that systematic inflammatory stimulation with LPS exacerbated the intensity of the ALO AIM score induced by the L-dopa treatment in the 6-OHDA-lesioned rats. To verify whether inhibiting LPS-induced inflammatory reaction may reduce the ALO AIM score in the 6-OHDA-lesioned rats treated with L-dopa, we used Clo-lipos to inhibit systemic inflammation induced by LPS. We found that the administration of Clo-lipo in the PD+Clo lipo+LPS+L-dopa group reduced the ALO AIM (Figure 6Aa), axial AIM (Figure 6Ab), limb AIM (Figure 6Ac), and orolingual AIM (Figure 6Ad) score compared with the PD+PBS lipo+LPS+L-dopa group (^*^p < 0.05, ^**^p < 0.01 vs. PD+Clo lipo+LPS+L-dopa, *n* = 12/group).

To evaluate whether the administration of Clo-lipo can attenuate peripheral LPS injection-induced neuroinflammation in the striatum, we used the immunohistochemical analysis and the Western blot of Iba-1 and GFAP to determine the microglia and astrocyte response in 21 days after the L-dopa treatment. The PD+PBS lipo+LPS+L-dopa group showed clear microglial and astrocyte activation compared with the PD+Clo lipo+LPS+L-dopa group (^**^p < 0.01, ^***^p < 0.001, vs. PD+ saline +L-dopa, ^#^*p* < 0.05, ^##^*p* < 0.01 vs. PD+PBS lipo+LPS+L-dopa, Figures 6B,C, *n* = 4/group). The administration of Clo-lipo can inhibit the release of neuroinflammatory cytokines IL-1β, IL-6, and TNF-α in the lesioned striatum compared with the PD+PBS lipo+LPS+L-dopa group (^***^p < 0.001, vs. PD+ saline +L-dopa, ^#^*p* < 0.05, vs. PD+PBS lipo+LPS+L-dopa, Figure 6D,*n* = 4/group).

### Clo-lipo Administration Prevents the Activation of Striatal NR2B-Mediated PKC/MEK/ERK and NF-κB Signaling Pathways Induced by Peripheral LPS Injection in LID Rats

**Figure 7:**
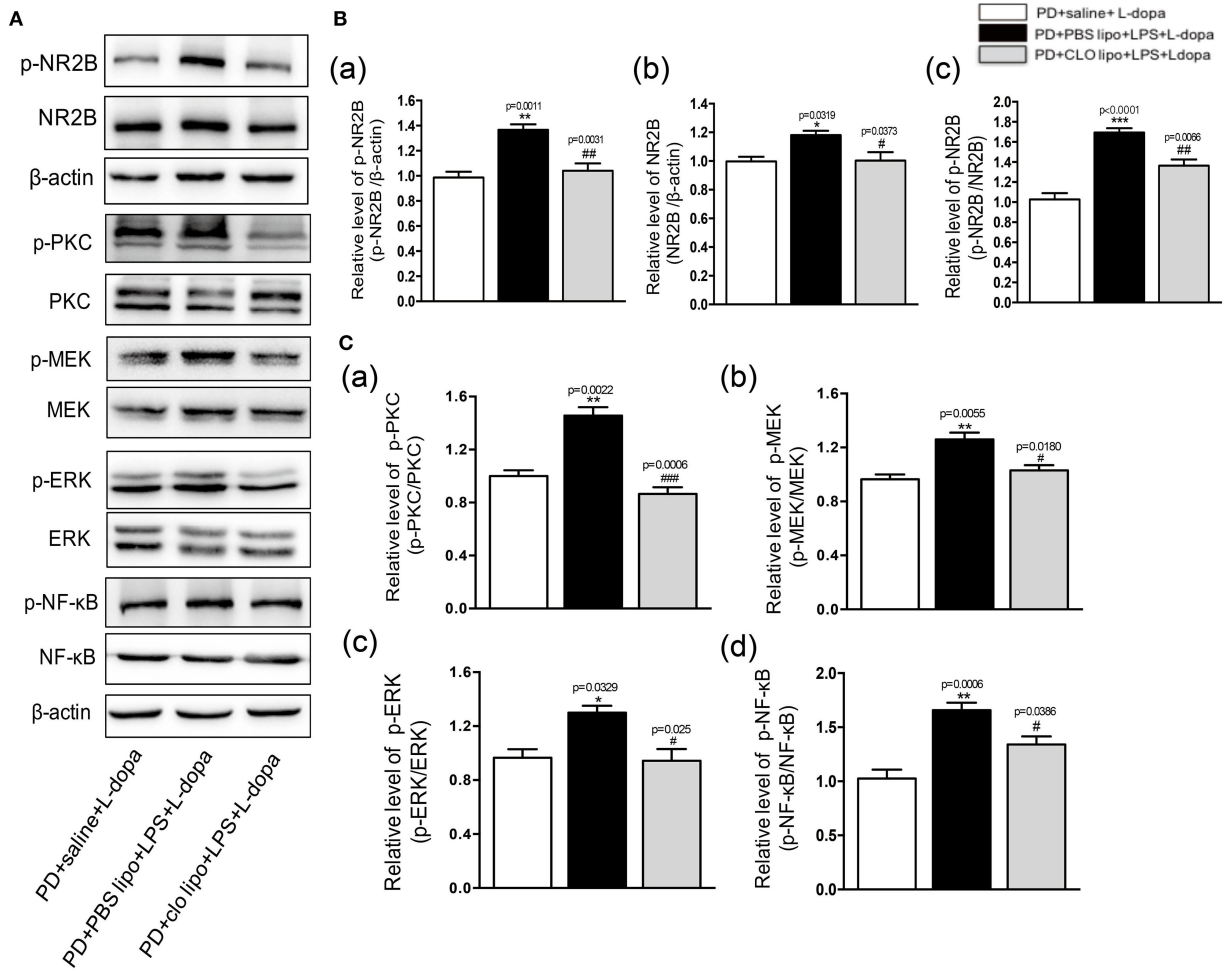
Effects of Clodronate liposome treatments on striatal NR2B, PKC/MEK/ERK, and NF-κB signaling pathways in LPS-treated LID rats. **(A)** Expression levels of p-NR2B, NR2B, p-PKC, PKC, p-MEK, MEK, p-ERK, ERK, p-NF-κB, and NF-κB in the striatum were analyzed by Western blotting. **(B)** (a) Quantification of the densitometric value of the p-NR2B protein bands is shown, normalized to β-actin. (b) Quantification of the densitometric value of the NR2B protein bands is shown, normalized to β-actin. (c) Quantification of the densitometric value of the p-NR2B protein bands is shown, normalized to NR2B. **(C)** (a) Quantification of the densitometric value of the p-PKC protein bands is shown, normalized to PKC. (b) Quantification of the densitometric value of the p-MEK protein bands is shown, normalized to MEK. (c) Quantification of the densitometric value of the p-ERK protein bands is shown, normalized to ERK. (d) Quantification of the densitometric value of the p-NF-κB protein bands is shown, normalized to NF-κB. Values are presented as mean ± SEM. *n* = 4 per group. *p < 0.05, **p < 0.01, and ***p < 0.001 vs. PD+saline+L-dopa. #*p* < 0.05, ##*p* < 0.01, ###*p* < 0.001, vs. PD+ PBS lipo+LPS+L-dopa.

In our study, the results showed that peripheral LPS injection markedly upregulated the striatal NR2B expression and activated its downstream PKC/MEK/ERK and NF-κB signaling pathways in the lesioned striatum as compared to the PD+saline+L-dopa group. Thus, we further examined the effects of Clo-lipo administration on the upregulation of p-NR2B and NR2B expression in the striatum of the LPS-treated LID rats. Firstly, we studied the effects of Clo-lipos on the LPS-treated rats. We found that the administration of Clo-lipos decreased LPS-stimulated p-NR2B and NR2B overexpression as compared to the PBS lipo+LPS group (Supplementary Figure 4). Furthermore, the Western blot analysis revealed that administration with Clo-lipos decreased p-NR2B and NR2B expression in the lesioned striatum of the PD+clo lipo+saline+L-dopa group as compared with the PD+PBS lipo+saline+L-dopa group (^*^p < 0.05, ^**^p < 0.01, ^***^p < 0.001, vs. PD+saline+L-dopa, ^#^*p* < 0.05, ^##^*p* < 0.01 vs. PD+PBS lipo+LPS+L-dopa, Figures 7A,B, *n* = 4/group). We next observed that the phosphorylation of PKC/MEK/ERK and NF-κB induced by peripheral LPS injection was largely inhibited by Clo-lipo administration as compared to the PD+PBS lipo+saline+L-dopa group (^*^p < 0.05, ^**^p < 0.01, vs. PD+saline+L-dopa, ^#^*p* < 0.05, ^###^*p* < 0.001 vs. PD+PBS lipo+LPS+L-dopa, Figures 7A,C, *n* = 4/group).

## Discussion

A growing body of studies demonstrated that the systemic inflammatory response could strongly influence the CNS (Holmes, 2013; Anthony and Couch, 2014). In our study, we used a single peripheral LPS injection to mimic the systemic inflammatory response to research the relationship between the periphery and the brain. Our study showed that systemic inflammation could increase the susceptibility to LID in 6-OHDA lesioned rats. Several findings are of interest in our study. Firstly, the peripheral LPS administration could induce neuroinflammation in the striatum of LID rats as demonstrated by the activation of glia cells and the increase in proinflammatory cytokines. Secondly, the exacerbation of LID in the LPS-induced systemic inflammation was also accompanied by the upregulation of both phosphorylated and total forms of NR2B in the striatum. Thirdly, systemic inflammation activated the PKC/MEK/ERK and NF-κB signaling pathways in LID rats. Clo-lipos ameliorated the effect of peripheral LPS injection, improved behavioral dysfunction, prevented NR2B overexpression, and decreased the phosphorylation of PKC/MEK/ERK and NF-κB signaling pathways.

There is increasing evidence that neuroinflammation may play a critical role in the etiogenesis of LID (Pisanu et al., 2018). Researches showed that inflammatory cytokines in the peripheral immune system might amplify neuroinflammation and contribute to the pathogenesis of a neurodegenerative disease (Perry et al., 2007; Cunningham et al., 2009; Ferrari and Tarelli, 2011). However, the role of systemic inflammation is unknown in LID. Thus, we wanted to study whether systemic inflammation would worsen the L-dopa-induced neuroinflammatory response in the striatum and exacerbate LID. To achieve this, systemic inflammation was induced by a single IP injection of LPS, an easy and well-characterized model of systemic inflammation. LPS is a major Gram-negative bacterial endotoxin that can trigger various cellular activities that contribute to the pathogenesis of inflammatory responses and induce the production of proinflammatory cytokines, such as TNF-α and IL-1β (Fu et al., 2014). Our study found that administration of LPS to the PD rats resulted in an increased susceptibility to the development of AIMs during the L-dopa treatment. In addition, several previous studies demonstrated that neuronal proinflammatory factors have been found to correlate with the LID expression in the 6-OHDA rat models of PD, where the development of AIM was accompanied by an increased immunoreactivity for the microglial marker OX-42, together with GFAP and with increases of nNOS, cyclooxygenase-2 (COX2), and NF-κB levels in the striatum (Bortolanza et al., 2015a,b; Dos-Santos-Pereira et al., 2016). Therefore, we analyzed the activation of striatal glial cells and the expression of pre-inflammatory cytokines in LPS-pretreated LID rats and controls. Our results revealed that LPS administration increased the number of activated microglia and astrocytes and promoted the pre-inflammatory cytokines TNF-a, IL-1β, and IL-6 in the striatum of LID rats.

Another striking finding of this study was the role of NR2B, a subunit of the NMDA receptor, in the LPS-treated LID rats. NMDARs are one of the major excitatory receptors within the CNS and have been the focus of potential therapeutics in a variety of neurological disorders (Yang et al., 2018). In the adult mammalian brain, NMDARs are comprised of different combinations of subunits of two classes, NR2A and NR2B, that are differentially distributed throughout the brain. Glycine confers neuroprotection through non-ionotropic activation of NR2A and subsequent enhancement of Akt activation (Zhang et al., 2018). On the other hand, the activation of NR2B plays an important role in the development and maintenance of chronic inflammatory response (Chen et al., 2008). NR2B was also found involved in the occurrence of LID (Blandini and Armentero, 2012). Many early studies performed in animals with dyskinetic conditions found an increase in the expression of NR2B in the striatum (Ahmed et al., 2011). NR2B antagonist CP-101,606 inhibits NR2B phosphorylation and provides benefit for the therapy of the LID rat model (Kong et al., 2015). A previous study showed that a single acute IP injection of LPS to postnatal male rats caused long-lasting (over 2 months) and possibly permanent changes in both the hippocampal and the cortex at the mRNA level of NR2B (subunit of NMDA; Harre et al., 2008). Our study found that the expression of phosphorylated and total forms of NR2B was markedly increased in the PD+LPS+L-dopa group compared with the PD+saline-L-dopa group. Thus, our findings confirmed that LPS-induced systemic inflammation could activate the NR2B submit. In addition, there is other evidence showing that peripheral inflammation can induce the activation of NR2B. For example, IL-1β can directly alter NR2B function *via* tyrosine kinase-mediated phosphorylation of NR2B (Viviani et al., 2003). NR2B subunits are overexpressed in an inflammatory model of Alzheimer's disease (AD; Maher et al., 2014). Therefore, we can speculate that a single IP injection of LPS would alter the NR2B subunit expression in the striatum of PD rats and lead to an increase in the susceptibility to LID in 6-OHDA lesioned rats. The downstream molecules resulting from NR2B activation may be responsible for behavioral deficits in the LPS-treated LID rats. Previous researches demonstrated that NMDA receptors could activate the PKC/ERK signaling pathway (Jia et al., 2007; Rodriguez-Duran and Escobar, 2014; Jiang et al., 2018), which may be due to positive coupling of the NR2B subunit to ERK (Sava et al., 2012). Furthermore, it was reported that hyperactivation of PKC/ERK and NF-κB by prolonged L-dopa treatment contributed to the development of LID (Bishnoi et al., 2008; Lin et al., 2017). Our results demonstrated that the administration of peripheral LPS could further increase the phosphorylation of PKC/MEK/ERK and NF-κB in LID rats.

Clodronate-liposome administration is a well-described method of depleting macrophages and reducing the experimental acute inflammatory response in an animal model. In the following experiment, we used Clo-lipos to ameliorate systemic inflammation induced by an LPS injection. Our results indicated that Clo-lipo administration significantly attenuated the AIM score of the LPS-treated LID rats. Furthermore, Clo-lipos decreased the LPS-induced activation of glial cells and the production of proinflammatory factors in the striatum. In addition, Clo-lipo administration reduced the overexpression of both phosphorylated and total forms of NR2B, which inhibited the phosphorylation of PKC/MEK/ERK and NF-κB signal pathways in the LPS-treated LID rats. These data suggested that Clo-lipos, which ameliorated the effect of LPS, improved behavioral dysfunction, prevented NR2B activation, and decreased the phosphorylation of PKC/MEK/ERK and NF-κB signaling pathways.

Our data indicated that systemic inflammation, by exacerbating neuroinflammation and facilitating the NR2B subunit activity, increased the susceptibility to LID in 6-OHDA lesioned rats, suggesting a possible pathophysiological role of the systemic inflammatory response in the appearance of dyskinesia, at least in the 6-OHDA-lesioned rat model of PD. On the other hand, the administration of Clo-lipo restored the effects of LPS, which further attenuated dyskinesia in the LPS-treated PD rats during the L-dopa treatment.

## Data Availability Statement

The original contributions presented in the study are included in the article/Supplementary Material, further inquiries can be directed to the corresponding author/s.

## Ethics Statement

The animal study was reviewed and approved by the Institutional Animal Care and Use Committee of the School of Medicine, Shanghai Jiao Tong University, Shanghai, China.

## Author Contributions

ZL and AY designed experiments. AY, LS, YZ, and XW performed the experiments. AY analyzed the results. AY wrote the manuscript with contributions from ZL. All authors read and approved the final manuscript.

## Conflict of Interest

The authors declare that the research was conducted in the absence of any commercial or financial relationships that could be construed as a potential conflict of interest.

## Supplementary Material

Supplementary Figure 1 Clodronate liposome treatment depleted macrophages in the spleen. **(A)** Immunohistochemistry analysis shows the CD68+ macrophages in the spleen of PD+PBS lipo and PD+CLP lipo groups at 3 days after liposome injection. **(B)** Bar graph shows quantification of the percentage of CD68+macrophages in the spleen of PD+PBS lipo and PD+CLP lipo groups.

Supplementary Figure 2 Effect of peripheral only one LPS injection on the striatal neuroinflammation of normal rats. **(A)** Expression levels of iba-1 (a,b) and GFAP (a,c) were analyzed by Western blotting. **(B)** The mRNA levels of the proinflammatory mediators IL-1β (a), IL-6 (b), and TNF-α (c) normalized to GAPDH in the striatum. Values are presented as mean ± SEM. *n* = 4 per group. ^*^*p* < 0.05, ^**^*p* < 0.01, and ^***^*p* < 0.001 vs. saline.

Supplementary Figure 3 LPS treatments increased the expressions of p-NR2B and NR2B in the striatum of normal rats. **(A)** Expression levels of p-NR2B, NR2B, and mGLUR5 in the striatum were analyzed by Western blotting. **(B)** (a) Quantification of the densitometric value of the p-NR2B protein bands is shown, normalized to β-actin. (b) Quantification of the densitometric value of the NR2B protein bands is shown, normalized to β-actin. (c) Quantification of the densitometric value of the p-NR2B protein bands is shown, normalized to NR2B. **(C)** Quantification of the densitometric value of the mGLUR5 protein bands is shown, normalized to β-actin. Values are presented as mean ± SEM. *n* = 4 per group. ^*^*p* < 0.05, ^**^*p* < 0.01, and ^***^*p* < 0.001 vs. saline.

Supplementary Figure 4 Effects of Clodronate liposome treatments on striatal p-NR2B, NR2B, and mGLUR5 in LPS-treated rats. **(A)** Expression levels of p-NR2B, NR2B, and mGLUR5 in the striatum were analyzed by Western blotting. **(B)** Quantification of the densitometric value of the p-NR2B protein bands is shown, normalized to β-actin. **(C)** Quantification of the densitometric value of the NR2B protein bands is shown, normalized to β-actin. **(D)** Quantification of the densitometric value of the mGLUR5 protein bands is shown, normalized to β-actin. Values are presented as the mean ± SEM. *n* = 4 per group. ^*^*p* < 0.05, ^**^*p* < 0.01, and ^***^*p* < 0.001 vs. PBS lipo+LPS. ^&&^*p* < 0.01, ^&&&^*p* < 0.001 vs. PBS lipo+saline.

## References

[B1] Ahmed I. Bose S. K. Pavese N. Ramlackhansingh A. Turkheimer F. Hotton G.. (2011). Glutamate NMDA receptor dysregulation in Parkinson's disease with dyskinesias. Brain 134 (Pt. 4), 979–986. 10.1093/brain/awr028 21371994

[B2] Anthony D. C. Couch Y. (2014). The systemic response to CNS injury. Exp. Neurol. 258, 105–111. 10.1016/j.expneurol.2014.03.013 25017891

[B3] Ba M. Ding W. Guan L. Lv Y. Kong M. (2019). S-nitrosylation of Src by NR2B-nNOS signal causes Src activation and NR2B tyrosine phosphorylation in levodopa-induced dyskinetic rat model. Hum. Exp. Toxicol. 38, 303–310. 10.1177/0960327118806633 30350722

[B4] Bishnoi M. Chopra K. Kulkarni S. K. (2008). Differential striatal levels of TNF-alpha, NFkappaB p65 subunit and dopamine with chronic typical and atypical neuroleptic treatment: role in orofacial dyskinesia. Prog. Neuropsychopharmacol. Biol. Psychiatry 32, 1473–1478. 10.1016/j.pnpbp.2008.05.003 18554768

[B5] Blandini F. Armentero M. T. (2012). New pharmacological avenues for the treatment of L-DOPA-induced dyskinesias in Parkinson's disease: targeting glutamate and adenosine receptors. Expert Opin. Investig. Drugs 21, 153–168. 10.1517/13543784.2012.651457 22233485

[B6] Bortolanza M. Cavalcanti-Kiwiatkoski R. Padovan-Neto F. E. da-Silva C. A. Mitkovski M. Raisman-Vozari R. (2015a). Glial activation is associated with l-DOPA induced dyskinesia and blocked by a nitric oxide synthase inhibitor in a rat model of Parkinson's disease. Neurobiol. Dis. 73, 377–387. 10.1016/j.nbd.2014.10.017 25447229

[B7] Bortolanza M. Padovan-Neto F. E. Cavalcanti-Kiwiatkoski R. Dos Santos-Pereira M. Mitkovski M. Raisman-Vozari R.. (2015b). Are cyclooxygenase-2 and nitric oxide involved in the dyskinesia of Parkinson's disease induced by L-DOPA? Philos. Trans. R. Soc. Lond. B. Biol. Sci. 370:20140190. 10.1098/rstb.2014.0190 26009769 PMC4455759

[B8] Carta A. R. Mulas G. Bortolanza M. Duarte T. Pillai E. Fisone G.. (2017). l-DOPA-induced dyskinesia and neuroinflammation: do microglia and astrocytes play a role? Eur. J. Neurosci 45, 73–91. 10.1111/ejn.13482 27859864

[B9] Chen L. Liu J. C. Zhang X. N. Guo Y. Y. Xu Z. H. Cao W.. (2008). Down-regulation of NR2B receptors partially contributes to analgesic effects of Gentiopicroside in persistent inflammatory pain. Neuropharmacology 54, 1175–1181. 10.1016/j.neuropharm.2008.03.007 18410946

[B10] Cho C. H. Kim J. Ahn J. Y. Hahn H. G. Cho S. W. (2015). N-adamantyl-4-methylthiazol-2-amine suppresses lipopolysaccharide-induced brain inflammation by regulating NF-kappaB signaling in mice. J. Neuroimmunol 289, 98–104. 10.1016/j.jneuroim.2015.10.016 26616878

[B11] Cilia R. Akpalu A. Sarfo F. S. Cham M. Amboni M. Cereda E.. (2014). The modern pre-levodopa era of Parkinson's disease: insights into motor complications from sub-Saharan Africa. Brain 137 (Pt. 10), 2731–2742. 10.1093/brain/awu195 25034897 PMC4163032

[B12] Cunningham C. Campion S. Lunnon K. Murray C. L. Woods J. F. Deacon R. M.. (2009). Systemic inflammation induces acute behavioral and cognitive changes and accelerates neurodegenerative disease. Biol. Psychiatry 65, 304–312. 10.1016/j.biopsych.2008.07.024 18801476 PMC2633437

[B13] Del-Bel E. Bortolanza M. Dos-Santos-Pereira M. Bariotto K. Raisman-Vozari R. (2016). l-DOPA-induced dyskinesia in Parkinson's disease: are neuroinflammation and astrocytes key elements? Synapse 70, 479–500. 10.1002/syn.21941 27618286

[B14] Dos-Santos-Pereira M. da-Silva C. A. Guimaraes F. S. Del-Bel E. (2016). Co-administration of cannabidiol and capsazepine reduces L-DOPA-induced dyskinesia in mice: Possible mechanism of action. Neurobiol. Dis. 94, 179–195. 10.1016/j.nbd.2016.06.013 27373843

[B15] Espay A. J. Morgante F. Merola A. Fasano A. Marsili L. Fox S. H.. (2018). Levodopa-induced dyskinesia in Parkinson disease: current and evolving concepts. Ann. Neurol. 84, 797–811. 10.1002/ana.25364 30357892

[B16] Ferrari C. C. Tarelli R. (2011). Parkinson's disease and systemic inflammation. Parkinsons. Dis. 2011:436813. 10.4061/2011/436813 21403862 PMC3049348

[B17] Fu Y. Y. Zhang F. Zhang L. Liu H. Z. Zhao Z. M. Wen X. R.. (2014). Mangiferin regulates interleukin-6 and cystathionine-b-synthase in lipopolysaccharide-induced brain injury. Cell. Mol. Neurobiol. 34, 651–657. 10.1007/s10571-014-0039-8 24794713 PMC11488922

[B18] Gan J. Qi C. Liu Z. (2015). Roles of Ca(2+)/calmodulin-dependent protein kinase II in subcellular expression of striatal N-methyl-D-aspartate receptors in l-3, 4-dihydroxyphenylalanine-induced dyskinetic rats. Drug Des. Devel. Ther. 9, 2119–2128. 10.2147/DDDT.S73868 25926720 PMC4403745

[B19] Gan J. Qi C. Mao L. M. Liu Z. (2014). Changes in surface expression of N-methyl-D-aspartate receptors in the striatum in a rat model of Parkinson's disease. Drug Des. Devel. Ther. 8, 165–173. 10.2147/DDDT.S51559 24465126 PMC3900317

[B20] Harre E. M. Galic M. A. Mouihate A. Noorbakhsh F. Pittman Q. J. (2008). Neonatal inflammation produces selective behavioural deficits and alters N-methyl-D-aspartate receptor subunit mRNA in the adult rat brain. Eur. J. Neurosci. 27, 644–653. 10.1111/j.1460-9568.2008.06031.x 18279317 PMC3547975

[B21] Herring W. J. Assaid C. Budd K. Vargo R. Mazenko R. S. Lines C.. (2017). A Phase Ib randomized controlled study to evaluate the effectiveness of a single-dose of the NR2B selective N-Methyl-D-aspartate antagonist MK-0657 on levodopa-induced dyskinesias and motor symptoms in patients with Parkinson disease. Clin. Neuropharmacol. 40, 255–260. 10.1097/WNF.0000000000000241 29059133

[B22] Ho Y. H. Lin Y. T. Wu C. W. Chao Y. M. Chang A. Y. Chan J. Y. (2015). Peripheral inflammation increases seizure susceptibility via the induction of neuroinflammation and oxidative stress in the hippocampus. J. Biomed. Sci. 22:46. 10.1186/s12929-015-0157-8 26100815 PMC4477313

[B23] Holmes C. (2013). Review: systemic inflammation and Alzheimer's disease. Neuropathol. Appl. Neurobiol. 39, 51–68. 10.1111/j.1365-2990.2012.01307.x 23046210

[B24] Jia J. Wang X. Li H. Han S. Zu P. Li J. (2007). Activations of nPKCepsilon and ERK1/2 were involved in oxygen-glucose deprivation-induced neuroprotection via NMDA receptors in hippocampal slices of mice. J. Neurosurg. Anesthesiol. 19, 18–24. 10.1097/01.ana.0000211020.88431.e2 17198096

[B25] Jiang S. Li X. Jin W. Duan X. Bo L. Wu J.. (2018). Ketamine-induced neurotoxicity blocked by N-Methyl-d-aspartate is mediated through activation of PKC/ERK pathway in developing hippocampal neurons. Neurosci. Lett. 673, 122–131. 10.1016/j.neulet.2018.02.051 29501685

[B26] Kalia L. V. Lang A. E. (2015). Parkinson's disease. Lancet 386, 896–912. 10.1016/S0140-6736(14)61393-3 25904081

[B27] Kong M. Ba M. Liu C. Zhang Y. Zhang H. Qiu H. (2015). NR2B antagonist CP-101,606 inhibits NR2B phosphorylation at tyrosine-1472 and its interactions with Fyn in levodopa-induced dyskinesia rat model. Behav. Brain Res 282, 46–53. 10.1016/j.bbr.2014.12.059 25576965

[B28] Kosyreva A. M. Makarova O. V. Kakturskiy L. V. Mikhailova L. P. Boltovskaya M. N. Rogov K. A. (2018). Sex differences of inflammation in target organs, induced by intraperitoneal injection of lipopolysaccharide, depend on its dose. J. Inflamm. Res 11, 431–445. 10.2147/JIR.S178288 30519071 PMC6233486

[B29] Lin J. Y. Liu Z. G. Xie C. L. Song L. Yan A. J. (2017). Antidyskinetic treatment with MTEP affects multiple molecular pathways in the parkinsonian striatum. Parkinsons. Dis. 2017:5798734. 10.1155/2017/5798734 29209553 PMC5682907

[B30] Ma Y. Li Y. Jiang L. Wang L. Jiang Z. Wang Y.. (2016). Macrophage depletion reduced brain injury following middle cerebral artery occlusion in mice. J. Neuroinflammation 13:38. 10.1186/s12974-016-0504-z 26873581 PMC4752808

[B31] Maher A. El-Sayed N. S. Breitinger H. G. Gad M. Z. (2014). Overexpression of NMDAR2B in an inflammatory model of Alzheimer's disease: modulation by NOS inhibitors. Brain Res. Bull 109, 109–116. 10.1016/j.brainresbull.2014.10.007 25454121

[B32] Mulas G. Espa E. Fenu S. Spiga S. Cossu G. Pillai E.. (2016). Differential induction of dyskinesia and neuroinflammation by pulsatile versus continuous l-DOPA delivery in the 6-OHDA model of Parkinson's disease. Exp. Neurol. 286, 83–92. 10.1016/j.expneurol.2016.09.013 27697481

[B33] Murta V. Farias M. I. Pitossi F. J. Ferrari C. C. (2015). Chronic systemic IL-1beta exacerbates central neuroinflammation independently of the blood-brain barrier integrity. J. Neuroimmunol 278, 30–43. 10.1016/j.jneuroim.2014.11.023 25595250

[B34] Perry V. H. Cunningham C. Holmes C. (2007). Systemic infections and inflammation affect chronic neurodegeneration. Nat. Rev. Immunol. 7, 161–167. 10.1038/nri2015 17220915

[B35] Pervin M. Karim M. R. Kuramochi M. Izawa T. Kuwamura M. Yamate J. (2018). Macrophage populations and expression of regulatory inflammatory factors in hepatic macrophage-depleted rat livers under lipopolysaccharide (LPS) treatment. Toxicol. Pathol. 46, 540–552. 10.1177/0192623318776898 29938593

[B36] Pisanu A. Boi L. Mulas G. Spiga S. Fenu S. Carta A. R. (2018). Neuroinflammation in L-DOPA-induced dyskinesia: beyond the immune function. J Neural Transm (Vienna). 10.1007/s00702-018-1874-4 29541852

[B37] Rodriguez-Duran L. F. Escobar M. L. (2014). NMDA receptor activation and PKC but not PKA lead to the modification of the long-term potentiation in the insular cortex induced by conditioned taste aversion: differential role of kinases in metaplasticity. Behav. Brain Res. 266, 58–62. 10.1016/j.bbr.2014.02.049 24631390

[B38] Sava A. Formaggio E. Carignani C. Andreetta F. Bettini E. Griffante C. (2012). NMDA-induced ERK signalling is mediated by NR2B subunit in rat cortical neurons and switches from positive to negative depending on stage of development. Neuropharmacology 62, 925–932. 10.1016/j.neuropharm.2011.09.025 22001284

[B39] Seiler P. Aichele P. Odermatt B. Hengartner H. Zinkernagel R. M. Schwendener R. A. (1997). Crucial role of marginal zone macrophages and marginal zone metallophils in the clearance of lymphocytic choriomeningitis virus infection. Eur. J. Immunol. 27, 2626–2633. 10.1002/eji.1830271023 9368619

[B40] Tronci E. Napolitano F. Munoz A. Fidalgo C. Rossi F. Bjorklund A.. (2017). BDNF over-expression induces striatal serotonin fiber sprouting and increases the susceptibility to l-DOPA-induced dyskinesia in 6-OHDA-lesioned rats. Exp. Neurol. 297, 73–81. 10.1016/j.expneurol.2017.07.017 28757258

[B41] Villaran R. F. Espinosa-Oliva A. M. Sarmiento M. De Pablos R. M. Arguelles S. Delgado-Cortes M. J.. (2010). Ulcerative colitis exacerbates lipopolysaccharide-induced damage to the nigral dopaminergic system: potential risk factor in Parkinson‘s disease. J. Neurochem. 114, 1687–1700. 10.1111/j.1471-4159.2010.06879.x 20584104

[B42] Viviani B. Bartesaghi S. Gardoni F. Vezzani A. Behrens M. M. Bartfai T.. (2003). Interleukin-1beta enhances NMDA receptor-mediated intracellular calcium increase through activation of the Src family of kinases. J. Neurosci. 23, 8692–8700. 10.1523/JNEUROSCI.23-25-08692.2003 14507968 PMC6740426

[B43] Wu K. L. Chan S. H. Chan J. Y. (2012). Neuroinflammation and oxidative stress in rostral ventrolateral medulla contribute to neurogenic hypertension induced by systemic inflammation. J. Neuroinflammation 9:212. 10.1186/1742-2094-9-212 22958438 PMC3462714

[B44] Xie C. L. Wang W. W. Zhang S. F. Yuan M. L. Che J. Y. Gan J.. (2014). Levodopa/benserazide microsphere (LBM) prevents L-dopa induced dyskinesia by inactivation of the DR1/PKA/P-tau pathway in 6-OHDA-lesioned Parkinson's rats. Sci. Rep. 4:7506. 10.1038/srep07506 25511986 PMC4267205

[B45] Xie X. Luo X. Liu N. Li X. Lou F. Zheng Y.. (2017). Monocytes, microglia, and CD200-CD200R1 signaling are essential in the transmission of inflammation from the periphery to the central nervous system. J. Neurochem. 141, 222–235. 10.1111/jnc.13972 28164283

[B46] Yan A. Zhang Y. Lin J. Song L. Wang X. Liu Z. (2018). Partial depletion of peripheral M1 macrophages reverses motor deficits in MPTP-treated mouse by suppressing neuroinflammation and dopaminergic neurodegeneration. Front. Aging Neurosci. 10:160. 10.3389/fnagi.2018.00160 29922149 PMC5996129

[B47] Yang L. Bai H. H. Zhang Z. Y. Liu J. P. Suo Z. W. Yang X.. (2018). Disruption of SHP1/NMDA receptor signaling in spinal cord dorsal horn alleviated inflammatory pain. Neuropharmacology 137, 104–113. 10.1016/j.neuropharm.2018.04.029 29758384

[B48] Zhang Z. Liu J. Fan C. Mao L. Xie R. Wang S.. (2018). The GluN1/GluN2B NMDA receptor and metabotropic glutamate receptor 1 negative allosteric modulator has enhanced neuroprotection in a rat subarachnoid hemorrhage model. Exp. Neurol. 301 (Pt. A), 13–25. 10.1016/j.expneurol.2017.12.005 29258835

