# Supplementary figures and images for: Systemic Inflammation Increases the Susceptibility to Levodopa-Induced Dyskinesia in 6-OHDA Lesioned Rats by Targeting the NR2B-Medicated PKC/MEK/ERK Pathway
Source: Front Aging Neurosci. 2021 Feb 1;12:625166. doi: 10.3389/fnagi.2020.625166 (PMC7882708; doi:10.3389/fnagi.2020.625166)

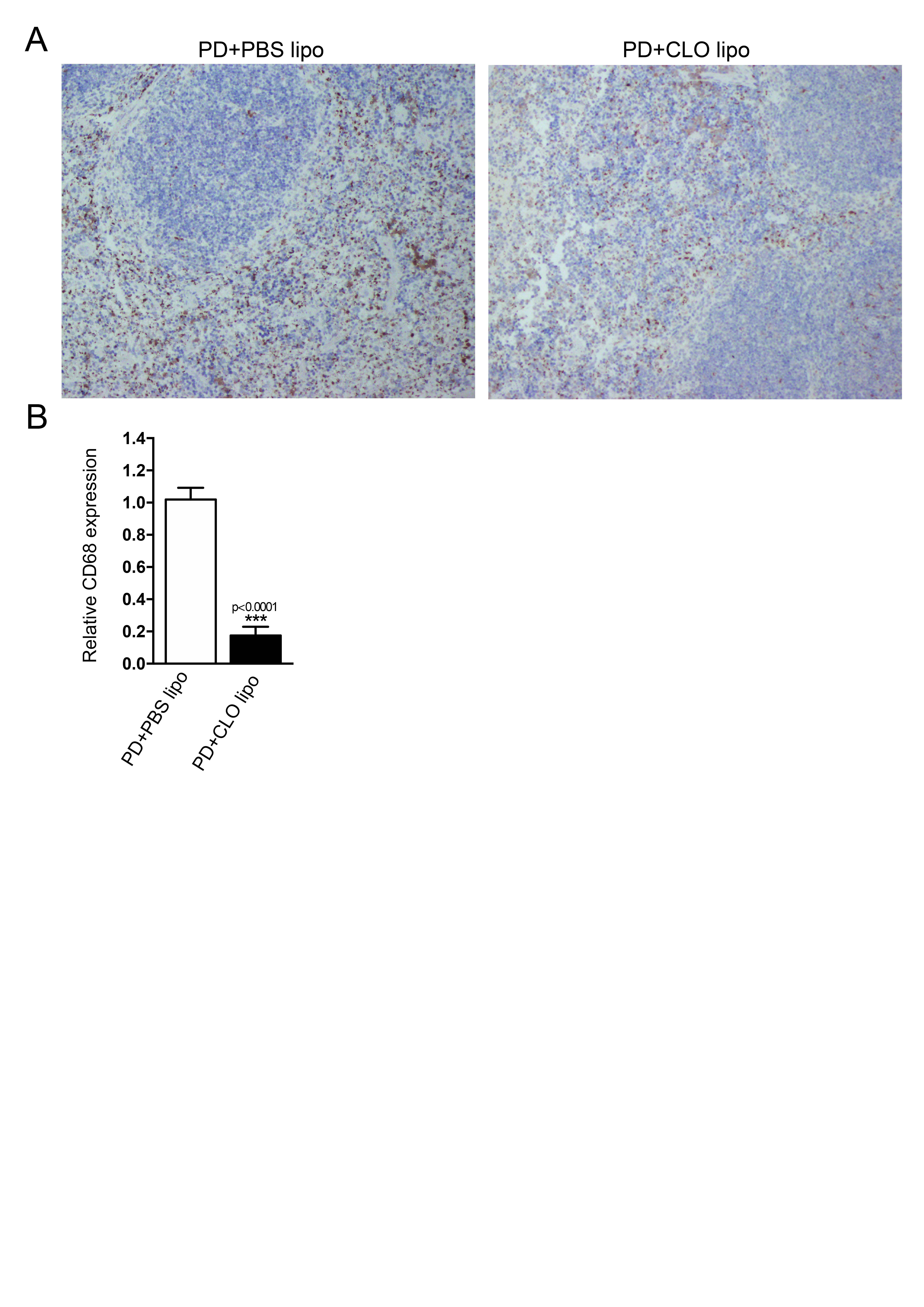

Supplement: Supplementary Figure 1 — Clodronate liposome treatment depleted macrophages in the spleen. (A) Immunohistochemistry analysis shows the CD68+ macrophages in the spleen of PD+PBS lipo and PD+CLP lipo groups at 3 days after liposome injection. (B) Bar graph shows quantification of the percentage of CD68+macrophages in the spleen of PD+PBS lipo and PD+CLP lipo groups. [file Image_1.JPEG]

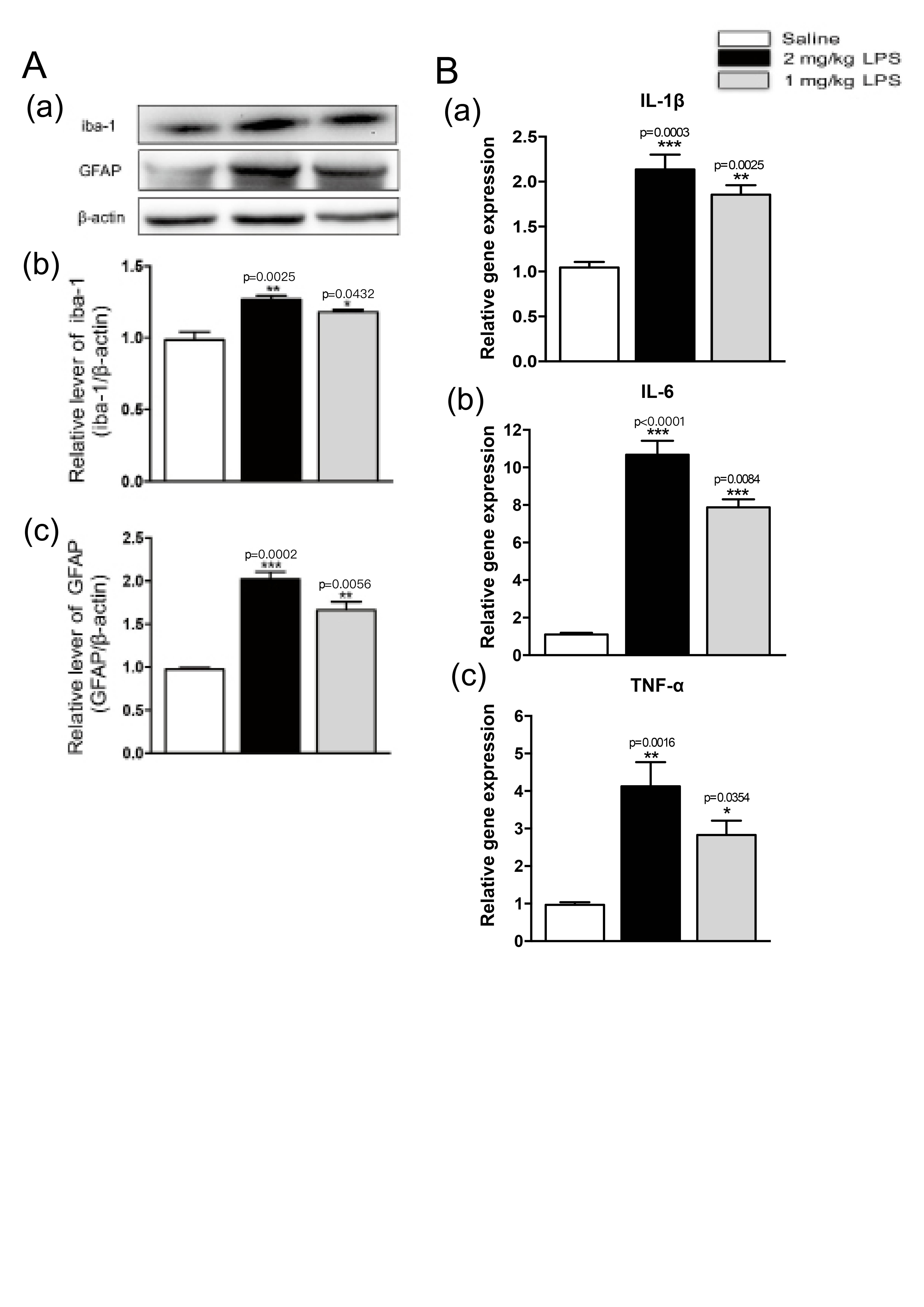

Supplement: Supplementary Figure 2 — Effect of peripheral only one LPS injection on the striatal neuroinflammation of normal rats. (A) Expression levels of iba-1 (a,b) and GFAP (a,c) were analyzed by Western blotting. (B) The mRNA levels of the proinflammatory mediators IL-1β (a), IL-6 (b), and TNF-α (c) normalized to GAPDH in the striatum. Values are presented as mean ± SEM. n = 4 per group. *p < 0.05, **p < 0.01, and ***p < 0.001 vs. saline. [file Image_2.JPEG]

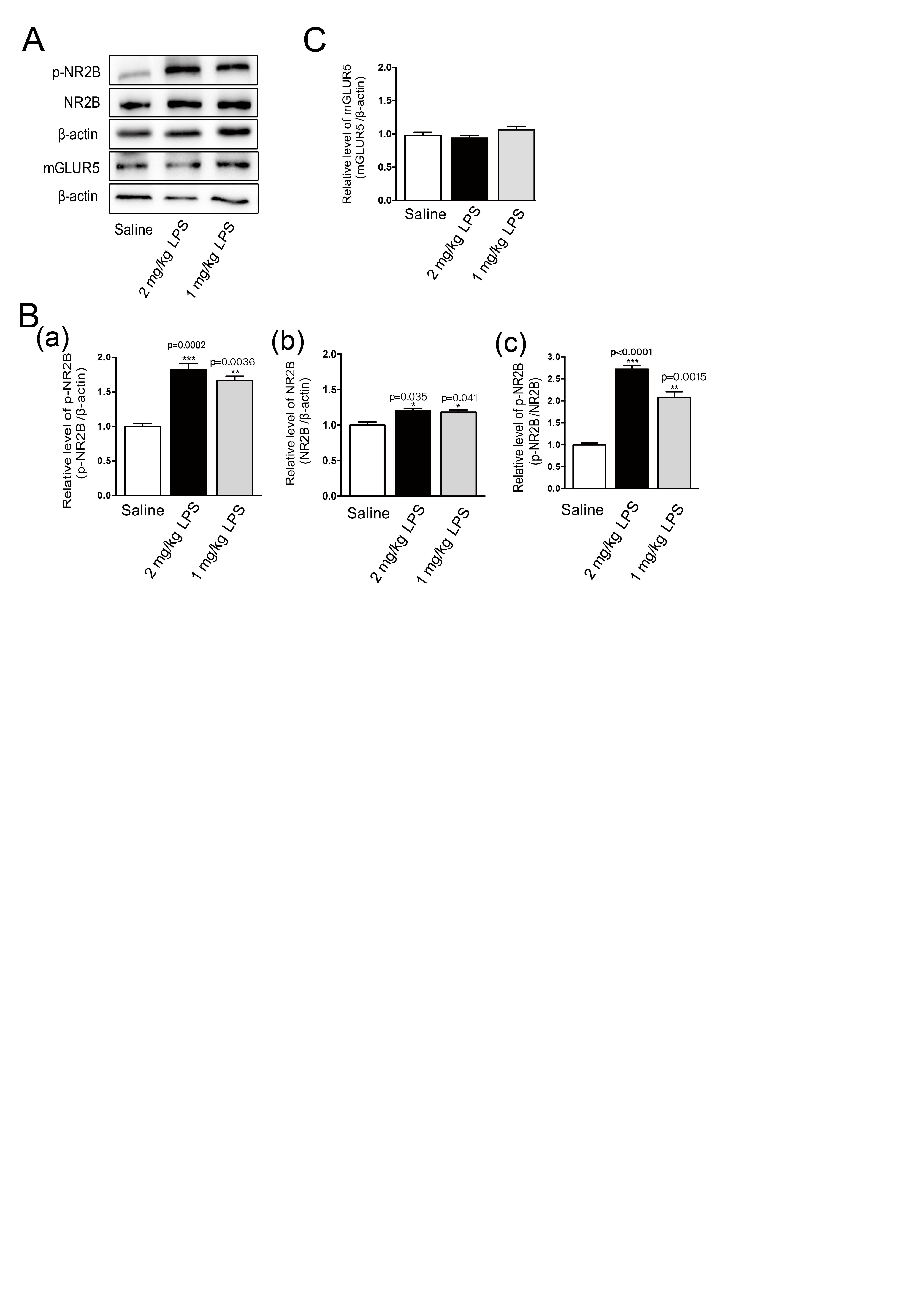

Supplement: Supplementary Figure 3 — LPS treatments increased the expressions of p-NR2B and NR2B in the striatum of normal rats. (A) Expression levels of p-NR2B, NR2B, and mGLUR5 in the striatum were analyzed by Western blotting. (B) (a) Quantification of the densitometric value of the p-NR2B protein bands is shown, normalized to β-actin. (b) Quantification of the densitometric value of the NR2B protein bands is shown, normalized to β-actin. (c) Quantification of the densitometric value of the p-NR2B protein bands is shown, normalized to NR2B. (C) Quantification of the densitometric value of the mGLUR5 protein bands is shown, normalized to β-actin. Values are presented as mean ± SEM. n = 4 per group. *p < 0.05, **p < 0.01, and ***p < 0.001 vs. saline. [file Image_3.JPEG]

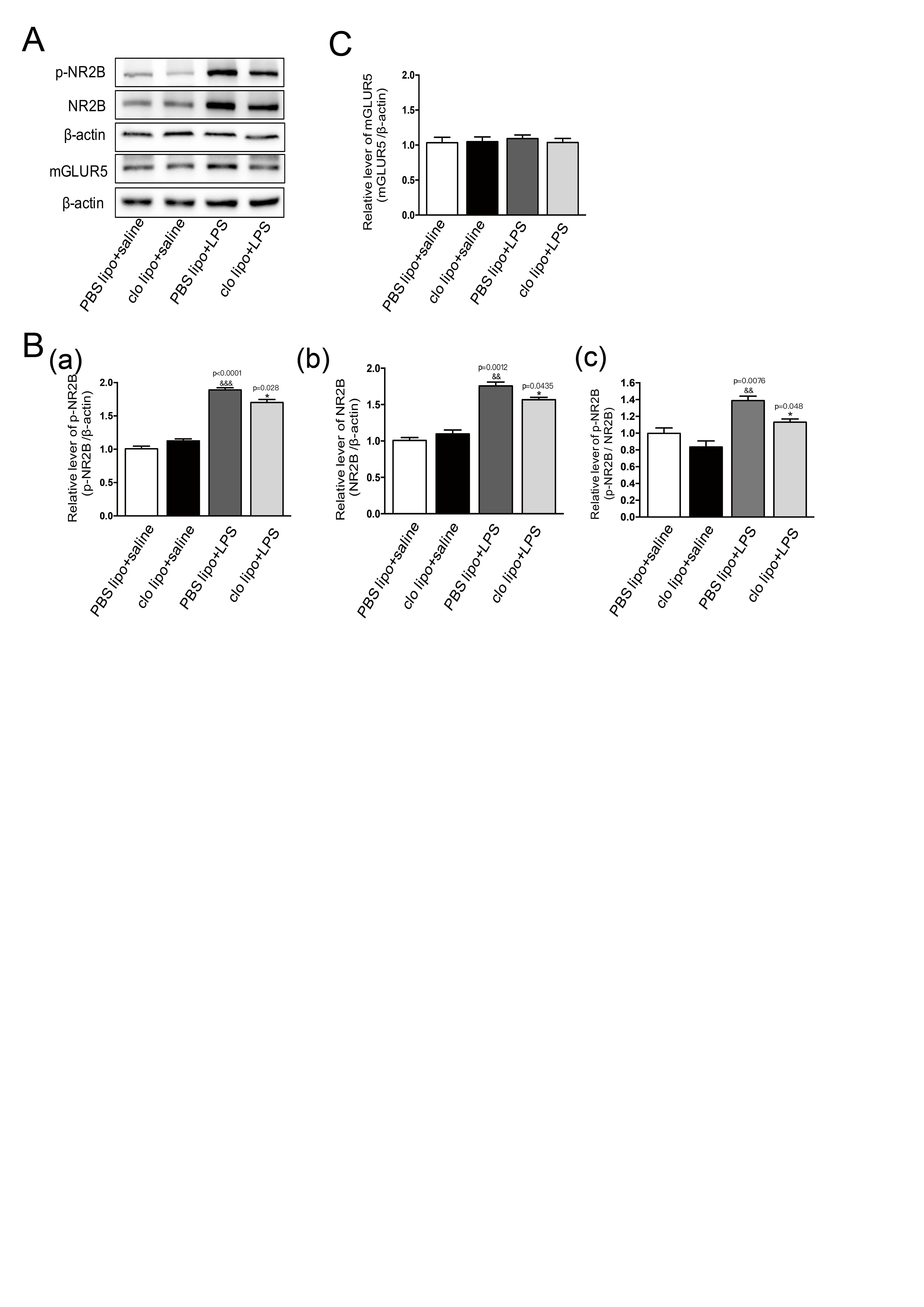

Supplement: Supplementary Figure 4 — Effects of Clodronate liposome treatments on striatal p-NR2B, NR2B, and mGLUR5 in LPS-treated rats. (A) Expression levels of p-NR2B, NR2B, and mGLUR5 in the striatum were analyzed by Western blotting. (B) Quantification of the densitometric value of the p-NR2B protein bands is shown, normalized to β-actin. (C) Quantification of the densitometric value of the NR2B protein bands is shown, normalized to β-actin. (D) Quantification of the densitometric value of the mGLUR5 protein bands is shown, normalized to β-actin. Values are presented as the mean ± SEM. n = 4 per group. *p < 0.05, **p < 0.01, and ***p < 0.001 vs. PBS lipo+LPS. &&p < 0.01, &&&p < 0.001 vs. PBS lipo+saline. [file Image_4.JPEG]
